# Clinical Utility of an Enzyme-Linked Immunosorbent Assay for Detecting Anti-Melanoma Differentiation-Associated Gene 5 Autoantibodies

**DOI:** 10.1371/journal.pone.0154285

**Published:** 2016-04-26

**Authors:** Shinji Sato, Akihiro Murakami, Akiko Kuwajima, Kazuhiko Takehara, Tsuneyo Mimori, Atsushi Kawakami, Michiaki Mishima, Takafumi Suda, Mariko Seishima, Manabu Fujimoto, Masataka Kuwana

**Affiliations:** 1 Division of Rheumatology, Department of Internal Medicine, Tokai University, School of Medicine, Isehara 259–1193, Japan; 2 Medical and Biological Laboratories, Nagoya 460–0008, Japan; 3 Department of Dermatology, Pharmaceutical and Health Sciences, Kanazawa University, Kanazawa 920–8641, Japan; 4 Department of Rheumatology and Clinical Immunology, Graduate School of Medicine, Kyoto University, Kyoto 606–8507, Japan; 5 Unit of Translational Medicine, Department of Immunology and Rheumatology, Nagasaki University Graduate School of Biomedical Sciences, Nagasaki 852–8501, Japan; 6 Department of Respiratory Medicine, Graduate School of Medicine, Kyoto University, Kyoto 606–8507, Japan; 7 Second Division, Department of Internal Medicine, Hamamatsu University School of Medicine, Hamamatsu 431–3192, Japan; 8 Department of Dermatology, Gifu University Graduate School of Medicine, Gifu 501–1194, Japan; 9 Department of Dermatology, Faculty of Medicine, Tsukuba University, Tsukuba 305–8575, Japan; 10 Department of Allergy and Rheumatology, Nippon Medical School Graduate School of Medicine, Tokyo 113–8603, Japan; Medical University of South Carolina, UNITED STATES

## Abstract

**Objective:**

Autoantibodies to melanoma differentiation-associated gene 5 (MDA5) are specifically expressed in patients with dermatomyositis (DM) and are associated with a subset of DM patients with rapidly progressive interstitial lung disease (RP-ILD). Here, we examined the clinical utility of a newly developed enzyme-linked immunosorbent assay (ELISA) system for detecting these antibodies.

**Methods:**

Here we developed an improved ELISA for detecting anti-MDA5 antibodies. We then performed a multicenter clinical study involving 8 medical centers and enrolled 242 adult patients with polymyositis (PM)/DM, 190 with non-PM/DM connective tissue disease (CTD), 154 with idiopathic interstitial pneumonia (IIP), and 123 healthy controls. Anti-MDA5 antibodies in the patients’ serum samples were quantified using our newly developed ELISA, and the results were compared to those obtained using the gold-standard immunoprecipitation (IP) assay. In addition, correlations between the ELISA-quantified anti-MDA5 antibodies and clinical characteristics were evaluated.

**Results:**

In patients with PM/DM, the anti-MDA5 antibody measurements obtained from the ELISA and IP assay were highly concordant; the ELISA exhibited an analytical sensitivity of 98.2%, specificity of 100%, positive predictive value of 100%, and negative predictive value of 99.5% (compared to the IP assay). Anti-MDA5 antibodies were detected in 22.7% of the DM patients, but not in any of the patients with PM, non-PM/DM CTD, or IIP. Clinically amyopathic DM, RP-ILD, arthritis, and fever were more prevalent in DM patients who were anti-MDA5 antibody-positive than in those who were antibody-negative (*P* ≤ 0.0002 for all comparisons). In addition, anti-MDA5 antibody-positive patients with RP-ILD exhibited higher antibody levels than those without RP-ILD (*P* = 0.006).

**Conclusion:**

Our newly developed ELISA can detect anti-MDA5 antibodies as efficiently as the gold standard IP assay and has the potential to facilitate the routine clinical measurement of anti-MDA5 antibodies in patients who suspected to have DM.

## Introduction

Circulating autoantibodies directed against nuclear or cellular components are commonly detected in patients with polymyositis (PM) or dermatomyositis (DM) [1]. In addition to well-characterized PM/DM-specific autoantibodies, such as anti-aminoacyl tRNA synthetase (ARS), anti-signal recognition particle, and anti-Mi-2 antibodies, a number of additional DM-specific antibodies have been recently described. These include antibodies against melanoma differentiation-associated gene 5 (MDA5), transcriptional intermediary factor-1-gamma, NXP-2, and small ubiquitin-like modifier activating enzyme [2]. The detection of these autoantibodies is highly useful for diagnosing PM/DM. Because of the strong associations of these autoantibodies with certain clinical characteristics of PM/DM, such as inflammatory myopathy, skin lesions, and interstitial lung disease (ILD), they are important biomarkers for classifying disease subgroups, predicting future organ involvement, and determining the prognosis of patients with PM/DM [1, 2].

Anti-MDA5 antibodies (also referred to as anti-CADM-140 antibodies) were first identified in the serum from patients with clinically amyopathic DM (CADM) by immunoprecipitation (IP) assays and shown to recognize a cytoplasmic 140-kDa protein [3]. The 140-kDa autoantigen was subsequently identified as MDA5, an RNA helicase, by molecular cloning techniques [4]. The production of anti-MDA5 antibodies is strongly associated with DM, especially with CADM, and rapidly progressive ILD (RP-ILD), and this subset is associated with particularly poor clinical outcomes [3–11]. There is currently no evidence-based treatment for RP-ILD in anti-MDA5 antibody-positive patients; however, intensive immunosuppressive therapy initiated early in the disease, before irreversible lung damage, may improve patient survival [2]. The early detection of anti-MDA5 antibodies helps to identify patients at high risk for developing life-threating RP-ILD. However, anti-MDA5 antibody measurement is not feasible in routine clinical laboratories, because the only accurate assay for detecting these antibodies is a complicated IP assay involving the use of a radioisotope and cultured cells [3]. Previously, two of us (SS and MK) developed an enzyme-linked immunosorbent assay (ELISA) for detecting anti-MDA5 antibodies that uses recombinant MDA5 as an antigen source [4]. This assay exhibits high analytical specificity (100%), but somewhat lower sensitivity (85%) than the gold standard IP assay. In the present study, we developed an improved version of the ELISA and examined its clinical utility using a multicenter study involving a large number of PM/DM patients and disease controls.

## Patients and Methods

### Patients and controls

We conducted a multicenter study at 8 medical centers across Japan from October 2011 to March 2014, and enrolled 242 adult patients with PM/DM, 190 with non-PM/DM connective tissue disease (CTD), and 154 with idiopathic interstitial pneumonia (IIP). The participants with PM/DM were consecutive patients at the individual medical centers, while the patients with non-PM/DM CTD or IIP were randomly selected from the outpatient population. Serum samples were collected in conjunction with retrospectively collected clinical information. PM/DM was defined as having definite or probable PM or DM according to the criteria proposed by Bohan and Peter [12], or having CADM according to the criteria proposed by Sontheimer; the presence of typical DM rashes for more than 2 years without clinical evidence of muscle symptoms [13]. The diagnoses of rheumatoid arthritis (RA), systemic lupus erythematosus (SLE), systemic sclerosis (SSc), and primary Sjögren’s syndrome were made according to the corresponding criteria proposed by the American College of Rheumatology [14–17]. Mixed connective tissue disease (MCTD) was defined according to published diagnostic criteria [18]. IIP was defined as ILD of unknown cause, including drug or occupational-environmental exposure, without fulfilling the classification criteria for any specific CTD or vasculitis [19]. Serum samples were collected from all of the patients and from 123 healthy control volunteers and stored at -20°C until use. Written informed consent was obtained from all of the study participants, in accordance with the Declaration of Helsinki. This study was approved by the Institutional Review Board of Tokai University, Kanazawa University, Kyoto University, Nagasaki University, Hamamatsu University, Gifu University, and Keio University.

### Clinical features

Demographic information, including sex and age at disease onset, was obtained from all patients. The clinical and laboratory findings of the PM/DM patients were retrospectively collected from medical records. The data obtained included PM/DM subgroup classification (PM, classic DM, and CADM) and the presence or absence of the following clinical features: heliotrope rash, Gottron’s sign, arthritis, fever, ILD, and malignancy. ILD was classified as RP-ILD or chronic ILD based on its progression during the disease course. Patients who exhibited a radiologic worsening of their ILD with progressive dyspnea and/or hypoxemia within 3 months of respiratory symptom onset were defined as having RP-ILD [4].

### IP assay

Serum samples from the study participants were subjected to IP assays using unlabeled or ^35^S-labeled HeLa cell extracts to analyze the RNA and protein components of autoantigens, respectively [3]. The sera were defined as anti-MDA5 antibody-positive when they reacted with a 140-kDa protein that was identical to that immunoprecipitated by the anti-MDA5 antibody-positive reference serum [3]. Anti-Jo-1, anti-EJ, anti-OJ, anti-PL-7, anti-PL-12, and anti-KS antibodies were identified by precipitation of the corresponding transfer RNA using RNA-IP assays.

### Preparation of recombinant human MDA5 antigen

A full-length complementary DNA encoding human MDA5 was kindly provided by Dr. Takashi Fujita (Kyoto University, Japan) [20]. Recombinant MDA5 protein was expressed as an N-terminal glutathione S-transferase (GST) fusion protein using a baculovirus expression system (BaculoGold™, BD Biosciences, San Jose, CA). Insect cells (High Five™; Thermo Fisher Scientific, Waltham, MA, USA) were infected with baculovirus harboring MDA5 and incubated for 72 hours at 27°C. The cells were lysed, and the soluble recombinant MDA5 was purified on a glutathione Sepharose 4B column (GE Healthcare, Buckinghamshire, UK) and eluted with 50 mM Tris-HCl (pH 8.0) containing 15 mM glutathione and 1.5 mM dithiothreitol (DTT). The purity of the recombinant MDA5 was examined by sodium dodecyl sulfate-12.5% polyacrylamide gel electrophoresis, followed by staining with 0.05% Coomassie blue and densitometric analysis with an Experia™ system (Bio-Rad Laboratories, Hercules, CA). The antigenicity of the recombinant MDA5 was analyzed on immunoblots probed with a rabbit anti-GST polyclonal antibody (Medical and Biological Laboratories, Nagoya, Japan), rabbit anti-MDA5 monoclonal antibody (Cell Signaling Technology, Danvers, MA), or anti-MDA5 antibody-positive or negative serum samples [4]. After incubating the membrane with peroxidase-conjugated anti-rabbit or anti-human IgG (Medical and Biological Laboratories), the immunoreactive bands were visualized using 3, 3’-diaminobenzidine tetrahydrochloride and hydrogen peroxide.

### Anti-MDA5 antibody ELISA

The anti-MDA5 antibody ELISA was developed primarily by applying a platform used for measuring anti-ARS antibodies (MESACUP anti-ARS test; Medical and Biological Laboratories) [21]. Briefly, purified recombinant MDA5 diluted to 4 μg/mL in phosphate-buffered saline (PBS) containing 2 mM DTT was coated onto 96-well Microtiter plates (Maxisorp; Nunc, Rochester, NY, USA) overnight at 4°C. The plates were washed twice with PBS and blocked with PBS containing 1% bovine serum albumin (BSA) and 5% sucrose overnight at 4°C. The serum samples were diluted 1:100 in PBS containing 0.5% sodium chloride, 0.15% Tween 20, 0.2% BSA, 1% casein enzymatic hydrolysate, and 0.2 mg/mL *Escherichia coli* extract, and incubated in the blocked plates for 30 minutes at room temperature. The plates were then washed four times with PBS containing 0.05% Tween 20 and incubated with peroxidase-conjugated anti-human IgG (Medical and Biological Laboratories) diluted 1:4,000 in 20 mM 4-(2-hydroxyethyl)-1-piperazineethanesulfonic acid, 135 mM sodium chloride, 1% BSA, and 0.1% p-hydroxyphenylacetic acid. After incubation for 30 minutes at room temperature, the plates were washed 4 times with PBS containing 0.05% Tween 20, and the bound antibodies were detected with the peroxidase substrate, 3, 3’, 5, 5’-tetramethylbenzidine. After incubation for 15 minutes at room temperature, the reaction was stopped by the addition of 0.5 N sulfuric acid. Absorbance at 450 nm (A_450_) was measured, and unit values (U/mL) were calculated from the following formula: 100 × (sample A_450_—blank A_450_) / (anti-MDA5-positive reference A_450_—blank A_450_).

### Detection of anti-ARS antibodies

Anti-ARS antibodies were detected using a commercial ELISA kit (Medical and Biological Laboratories). This system detects anti-Jo-1, anti-PL-7, anti-EJ, anti-PL-12, and anti-KS antibodies, but does not detect anti-OJ antibody [21]. The presence of individual anti-ARS antibodies was further confirmed by RNA-IP assays.

### Statistical analysis

The cut-off value that best discriminated two groups was determined by receiver operating characteristic (ROC) curve analysis. All continuous values are shown as the mean ± standard deviation. Unpaired comparisons of continuous variables were performed using the Mann-Whitney *U* test. Categorical variables were compared using a *chi*-square test or Fisher’s exact test when appropriate. Pairwise comparison was performed when the 2×3 table *chi*-square results were statistically significant. Statistical analysis was performed using StatView version 5.0 software (Artech, Osaka, Japan).

## Results

### Clinical characteristics of the enrolled patients

The 242 patients with PM/DM consisted of 70 with PM, 104 with classic DM, and 68 with CADM. CADM accounted for 40% of the consecutive cohort of total DM patients. This is consistent with the proportion of CADM in independent DM cohorts previously reported in Japan, ranging form 36% to 43% [3, 5, 22, 23]. The age at disease onset was 55.5 ± 15.1 years, and 169 patients (70%) were women. ILD was found in 134 of the PM/DM patients (57%), of which 65 (27%) had RP-ILD and 69 (29%) had chronic ILD. Malignancy was reported in 51 of the patients (21%). The patients with non-PM/DM CTD included 67 with SLE, 45 with RA, 43 with SSc, 20 with MCTD, 8 with primary Sjögren’s syndrome, 4 with overlapping syndrome, and 3 with miscellaneous diseases, such as inclusion body myositis, polymyalgia rheumatica, and anti-neutrophil cytoplasmic antibody-associated vasculitis. The patients with IIP consisted of 18 (12%) with RP-ILD and 136 (88%) with chronic ILD.

### Establishment of an anti-MDA5 antibody ELISA

**Fig 1:**
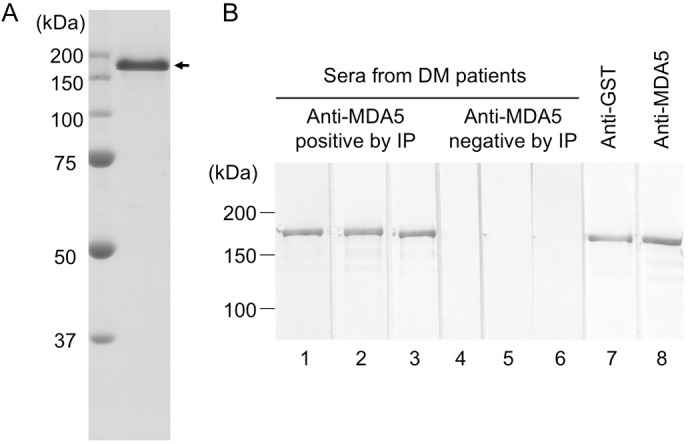
Purity and antigenicity of recombinant GST-MDA5 protein. **A.** Purified recombinant GST-MDA5 fusion protein (2 μg) was subjected to sodium dodecyl sulfate-12.5% polyacrylamide gel electrophoresis, followed by 0.05% Coomassie blue staining. The GST-MDA5 fusion protein was detected as a single band at ~166 kDa (arrow). **B.** Immunoblot analysis of the recombinant fusion protein was performed by probing with anti-MDA5 antibody-positive DM sera (lanes 1–3), anti-MDA5 antibody-negative DM sera (lanes 4–6), an anti-GST polyclonal antibody (lane 7), and an anti-MDA5 monoclonal antibody (lane 8).

A recombinant GST-MDA5 fusion protein was expressed and purified using a baculovirus expression system. The purity of the isolated protein was determined by Coomassie blue staining followed by densitometry, and shown to be >95% (Fig 1A). To assess the antigenicity of the GST-MDA5 protein, it was subjected to immunoblot analysis using 6 DM serum samples, 3 of which were anti-MDA5 antibody-positive and 3 of which were anti-MDA5 antibody-negative, as determined by IP assay (Fig 1B). As expected, the GST-MDA5 protein was specifically recognized by anti-MDA5 antibody-positive sera as well as by anti-GST and commercial anti-MDA5 antibodies. Faint ladder bands potentially corresponding to degradation products of the fusion protein were visible with antibodies that recognized MDA5, but not with the anti-GST antibody, suggesting that degradation primarily occurred in the GST portion of the fusion protein, and should not influence antigenicity.

**Fig 2:**
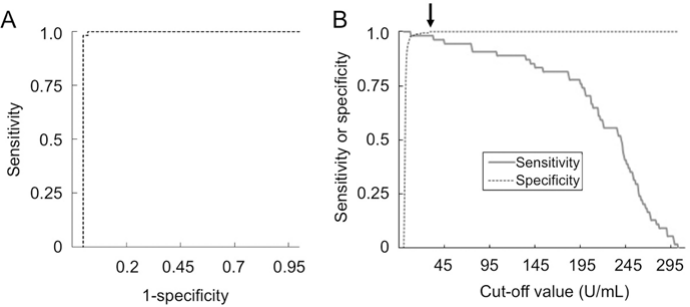
ROC curve analysis to determine the optimal cut-off value for ELISA-quantified anti-MDA5 antibodies. **A.** The ROC curve showed high concordance between the ELISA and IP assay (area under the curve > 0.99, *P* < 0.0001). **B.** The sensitivity and specificity of the anti-MDA5 antibody ELISA for various cutoff levels. A cutoff of 32 U/mL (arrow) provided a sensitivity of 98.2% and specificity of 100%.

We next developed an ELISA for detecting anti-MDA5 antibodies using the purified GST-MDA5 fusion protein as an antigen source. We evaluated the efficiency of the ELISA by using it to analyze the 242 PM/DM sera. In addition, all of the PM/DM sera were analyzed by the gold standard anti-MDA5 antibody IP assay. The IP assay showed that 56 of the serum samples (23.1%) reacted with a 140-kDa protein corresponding to MDA5. In close agreement, the anti-MDA5 antibody ELISA indicated that 55 of the samples (22.7%) were anti-MDA5 antibody-positive (Table 1). An ROC analysis was conducted to determine the optimal cut-off level of the ELISA-quantified anti-MDA5 antibody that differentiated between the anti-MDA5 antibody-positive and negative sera, as determined by the IP assay (Fig 2A). This analysis confirmed that the ELISA and IP assay results were highly concordant (area under the curve > 0.99, *P* < 0.0001). As shown in Fig 2B, an anti-MDA5 antibody level > 32 U/mL was determined to be the optimal cut-off for predicting positive results in the anti-MDA5 antibody IP assay, with an analytical sensitivity of 98.2%, specificity of 100%, positive predictive value of 100%, and negative predictive value of 99.5%. The analysis of only one serum resulted in a false-negative result in the ELISA. This serum was obtained from a patient who had received immunosuppressive treatment before blood collection, which may have caused the level of anti-MDA5 antibodies to fall to a level that was undetectable by the ELISA. Nevertheless, these results indicated that our newly developed anti-MDA5 antibody ELISA is a sensitive and specific method that has the potential to replace the gold standard IP assay.

**Table 1:** Frequencies of anti-MDA5 and anti-ARS antibodies detected by ELISA.

Diagnosis	Number of samples	Anti-MDA5 antibody	Anti-ARS antibody*	*P*
PM/DM, overall	242	55 (22.7%)	54 (22.3%)	1.0
PM	70	0	21 (30.0%)	< 0.0001
Classic DM	104	10 (9.6%)	30 (28.8%)	0.0008
CADM	68	45 (66.2%)	3 (4.4%)	< 0.0001
Non-PM/DM CTD	190	0	5 (2.6%)	0.07
IIP	154	0	14 (9.1%)	0.0003
Healthy controls	123	0	0	1.0

*This assay is able to detect anti-Jo-1, anti-EJ, anti-PL-7, anti-PL-12, and anti-KS antibodies, but does not detect anti-OJ antibody.

### Screening of anti-MDA5 and anti-ARS antibodies in the study participants

**Fig 3:**
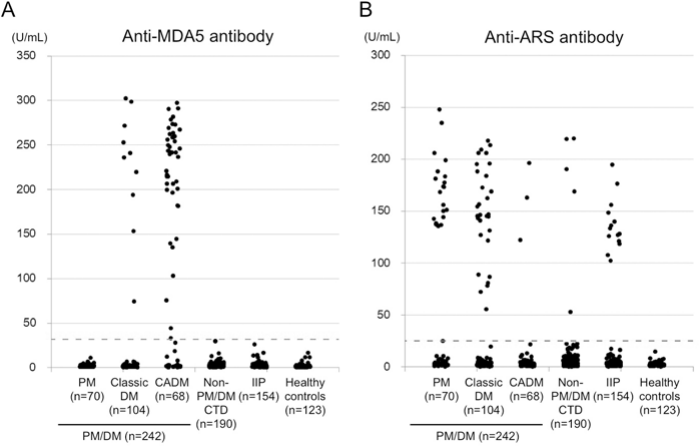
**Anti-MDA5 (A) and anti-ARS (B) antibody levels in patients with PM/DM, non-PM/DM CTD, IIP, and healthy controls.** Anti-MDA5 and anti-ARS antibodies were measured by ELISA in the sera from 242 patients with PM/DM (70 PM, 104 classic DM, and 68 CADM), 190 patients with non-PM/DM CTD, 154 patients with IIP, and 123 healthy volunteers. Cutoff levels of anti-MDA5 and anti-ARS antibodies are shown as broken lines (32 and 25 U/mL, respectively).

Next, we analyzed the anti-MDA5 antibody levels in the serum samples from 190 patients with non-PM/DM CTD, 154 patients with IIP, and 123 healthy controls using the anti-MDA5 antibody ELISA (Fig 3A). Notably, anti-MDA5 antibodies were not detected in any of these samples, including those from 18 patients with RP-ILD in the IIP group (**Table 1**). Interestingly, of the 55 (22.7%) PM/DM patients who were anti-MDA5 antibody-positive, only those patients diagnosed with either classic DM or CADM, but not PM, were anti-MDA5 antibody-positive. The clinical sensitivity and specificity for the diagnosis of DM, including classic DM and CADM, was 32.0% and 100%, respectively. Moreover, the positive and negative predictive values were 100% and 99.5%, respectively.

We also quantified the anti-ARS antibody levels in the serum samples of all of the study participants (Fig 3B). Anti-ARS antibodies were detected in patients with PM/DM, and in small numbers of patients with non-PM/DM CTD or IIP (Table 1), including 3 with SSc, and one each with SLE and MCTD. The overall clinical sensitivity and specificity of the anti-ARS ELISA for PM/DM was 22.3% and 94.5%, respectively. The prevalence of anti-MDA5 and anti-ARS antibodies in patients with PM/DM was almost identical, but their distribution across the PM/DM subgroups was quite different. While the anti-MDA5 antibodies were preferentially detected in patients with CADM, the anti-ARS antibodies were predominantly found in patients with PM or classic DM. Although we detected both types of antibodies in 2 patients with classic DM by ELISA analysis, it is likely that the anti-ARS antibody ELISA results in these cases were false positives, since the corresponding IP assays failed to confirm the presence of anti-ARS antibodies in these sera. Thus, our findings suggested that the presence of anti-MDA5 and anti-ARS antibodies was mutually exclusive in our study participants. The combined clinical sensitivity of the 2 antibodies was 44.2% in overall PM/DM patients; 30.0% in PM patients, 36.5% in classic DM patients, and 70.6% in CADM patients.

### Clinical features associated with anti-MDA5 antibodies detected by ELISA in patients with DM

Since anti-MDA5 antibodies were detected exclusively in patients with DM, we examined the clinical features associated anti-MDA5 antibody positivity in 172 patients with DM, including the classic DM and CADM patients. We divided the DM patients into 3 groups: patients who were anti-MDA5 antibody-positive, patients who were anti-ARS antibody-positive, and patients who were negative for both antibodies (antibody-negative patients). We then compared the demographic and clinical findings among the 3 groups (Table 2). We found that the age at initial examination was lower in patients with anti-MDA5 antibodies and tended to be lower in those with anti-ARS antibodies, compared with that of antibody-negative patients (*P* = 0.005 and *P* = 0.06, respectively). Classic DM was less frequent and CADM was more frequent in anti-MDA5 antibody-positive patients than in anti-ARS-positive or antibody-negative patients (*P* < 0.0001 for both comparisons). The overall frequency of ILD was similar between the anti-MDA5-positive and anti-ARS-positive groups, and was significantly greater than that of the antibody-negative group (*P* < 0.0001 for both comparisons). RP-ILD was more common in the anti-MDA5-positive group, while chronic ILD was more frequent in the anti-ARS-positive group, compared with the other 2 groups (*P* < 0.0001 for all comparisons). Therefore, anti-MDA5 antibody detection was useful for predicting RP-ILD development in patients with DM, with a sensitivity of 84%, specificity of 86%, positive predictive value of 73%, and negative predictive value of 92%. We also found that the anti-MDA5 antibody levels were significantly higher in anti-MDA5 antibody-positive DM patients with RP-ILD than in those without RP-ILD (231.0 ± 55.6 versus 168.6 ± 79.7, *P* = 0.006).

**Table 2:** Clinical characteristics of patients with DM, stratified by autoantibody status.

Demographic and clinical findings	Anti-MDA5 positive*	Anti-ARS positive*	Anti-MDA5/anti-ARS negative	*P*†
	(n = 55)	(n = 31)	(n = 86)	
Age at onset, mean ± SD	50.8 ± 14.3	52.9 ± 15.7	58.4 ± 17.3	0.01
Female, %	72.7	83.9	66.3	0.17
DM subgroup				
Classic DM, %	18.2	90.3	76.7	< 0.0001
CADM, %	81.8	9.7	23.3	< 0.0001
ILD overall, %	90.9	80.6	27.9	< 0.0001
ILD type				
RP-ILD, %	83.6	25.8	10.5	< 0.0001
Chronic ILD, %	7.3	54.8	17.4	< 0.0001
Gottron’s sign, %	87.3	67.7	79.1	0.10
Heliotrope rash, %	49.1	25.8	55.8	0.02
Arthritis, %	65.5	48.4	22.1	< 0.0001
Fever, %	61.8	35.5	30.2	0.0008
Malignancy, %	5.5	9.7	34.9	< 0.0001

*Two patients who were positive for both anti-MDA5 and anti-ARS by ELISA, but were negative for anti-ARS by IP assay were included in the anti-MDA5-positive group.

†Statistical analysis was performed by *chi*-square tests on a 2 × 3 table.

Heliotrope rash was more prevalent in the anti-MDA5-positive than in the anti-ARS-positive group (*P* = 0.02), while arthritis was more common in the anti-MDA5-positive group than in the antibody-negative group (*P* < 0.0001). Fever was more commonly found in anti-MDA5-positive patients than in anti-ARS-positive or antibody-negative patients (*P* = 0.01 and *P* = 0.0002, respectively). Finally, malignancy was less frequent in anti-MDA5 or anti-ARS antibody-positive patients than in antibody-negative patients (*P* < 0.0001 and *P* = 0.007, respectively).

When clinical characteristics were compared between DM patients with and without anti-MDA5 antibodies, 55 patients positive for anti-MDA5 antibodies represented younger disease onset (*P* = 0.01); higher frequency of CADM (*P* < 0.0001), ILD (*P* < 0.0001), RP-ILD (*P* < 0.0001), arthritis (*P* < 0.0001), and fever (*P* = 0.0002); and lower frequency of classic DM (*P* < 0.0001), chronic ILD (*P* = 0.002), and malignancy (*P* = 0.0005), compared with 117 negative for anti-MDA5 antibodies.

The majority of anti-MDA5 antibody-positive DM patients were classified as having CADM, although there were 10 patients positive for anti-MDA5 antibodies had classic DM. To further examine if associated clinical features in patients with CADM and classic DM were different, clinical characteristics were compared between classic DM patients with anti-MDA5 and anti-ARS antibodies (Table 3). Even within patients with classic DM, anti-MDA5 antibody was associated with RP-ILD and fever, while anti-ARS antibody was associated with chronic ILD.

**Table 3:** Comparisons of clinical characteristics in classic DM patients with anti-MDA5 and anti-ARS antibodies.

Demographic and clinical findings	Anti-MDA5 positive*	Anti-ARS positive*	*P*
	(n = 10)	(n = 28)	
Age at onset, mean ± SD	55.4 ± 14.8	54.3 ± 15.8	0.83
Female, %	60.0	82.1	0.21
ILD overall, %	80.0	85.7	0.64
ILD type			
RP-ILD, %	70.0	25.0	0.02
Chronic ILD, %	10.0	53.6	0.02
Gottron’s sign, %	90.0	67.9	0.24
Heliotrope rash, %	60.0	28.6	0.13
Arthritis, %	60.0	50.0	0.72
Fever, %	80.0	35.7	0.03
Malignancy, %	10.0	10.7	1.00

*Two patients who were positive for both anti-MDA5 and anti-ARS by ELISA, but were negative for anti-ARS by IP assay were included in the anti-MDA5-positive group.

## Discussion

In this study, we verified the efficiency of a newly developed anti-MDA5 antibody ELISA in a multi-center study involving patients with various connective tissue diseases or those with IIP. The new ELISA uses highly purified recombinant MDA5 protein as the antigen source and a platform that was previously validated in the commercial anti-ARS antibody detection kit; these features represent improvements over the original ELISA [4]. As a result, the new system achieved an analytical sensitivity and specificity that were comparable to those of the gold standard IP assay. Therefore, the new anti-MDA5 antibody ELISA has the potential to replace the IP assay, which is a complicated and time-consuming procedure that is currently performed only in some research laboratories. Since the ELISA can be used to screen large numbers of sera quickly and efficiently, it would be suitable for detection of anti-MDA5 antibodies in clinical practice.

Using the new ELISA, we showed that anti-MDA5 antibodies were highly specific to patients with DM, in particular to patients with CADM, and was not detected in patients with PM. Notably, we did not detect these antibodies in any of the non-PM/DM CTD or IIP patients. The strong association of anti-MDA5 antibody positivity with DM is consistent with previous reports [3, 4, 7–11]. In contrast, anti-ARS antibodies were detected in patients with various CTDs and even in patients with IIP, but were more prevalent in patients with PM/DM. Because of the mutually exclusive expression of anti-MDA5 and anti-ARS antibodies, the simultaneous measurement of both autoantibodies is predicted to improve the clinical diagnosis of PM/DM. In this regard, both anti-MDA5 and anti-ARS antibodies produce cytoplasmic staining by indirect immunofluorescence in routine autoantibody screening.

The majority of anti-MDA5 antibody-positive patients in this study lacked clinical findings indicative of inflammatory myopathy, and were diagnosed with CADM. This finding is consistent with those of previously published studies [3–8, 10], including a meta-analysis that demonstrated strong associations of anti-MDA5 antibodies with CADM in Japanese adult patients, and with classic DM in North American, Chinese, and Korean patients [10]. In the same study, the sensitivity and specificity of anti-MDA5 antibody detection for identifying RP-ILD in PM/DM patients were shown to be 77% and 86%, respectively. Our anti-MDA5 ELISA showed a similar level of performance in diagnosing RP-ILD in DM patients, suggesting that this assay system should be useful for detecting RP-ILD in the early phases of the disease and in making treatment decisions. Since the anti-MDA5 antibodies were never detected in IIP patients who did not exhibit DM manifestations, even though 18 of them had been diagnosed with RP-ILD, the production of these antibodies appears to be tightly associated with RP-ILD in the context of DM, but not with RP-ILD alone.

Anti-MDA5 antibodies detected by our new ELISA were associated with arthritis, fever, and lack of malignancy, in addition to CADM and RP-ILD. These clinical associations were principally consistent with previous studies [3, 6, 7, 24–27]. In addition, it has been reported that ulcerations and palmer papules are characteristic skin manifestations in patients with anti-MDA5 antibodies [7, 8, 25], but these clinical features was not recorded in our cohort. This is one of limitations of this study.

One of the advantages of the ELISA over the IP assay is that it provides quantitative results. We previously conducted a longitudinal analysis of anti-MDA5 antibody levels in DM patients using the previous version of the ELISA [28, 29], and found that high levels of anti-MDA5 antibodies at diagnosis were useful for predicting poor outcomes. In addition, we reported that the reduction and subsequent disappearance of antibodies during the course of immunosuppressive treatment was associated with favorable outcomes. The present study, involving a larger number of patients, confirmed the correlation between high antibody levels and RP-ILD. Taken together, these findings suggest that the anti-MDA5 antibody ELISA will be useful not only for early diagnosis and timely prediction of RP-ILD development, but also for monitoring disease activity and evaluating therapeutic efficacy.

In summary, we have shown that our newly developed anti-MDA5 antibody ELISA exhibited efficacy that was equivalent to that of the gold standard IP assay. The incorporation of this assay into routine clinical testing has the potential to improve the outcomes of patients with DM and RP-ILD by facilitating early diagnosis, timely intervention, and proper monitoring of disease activity.

## References

[1] Mimori T, Imura Y, Nakashima R, Yoshifuji H. Autoantibodies in idiopathic inflammatory myopathy: an update on clinical and pathophysiological significance. Curr Opin Rheumatol. 2007;19: 523–529. 17917530 10.1097/BOR.0b013e3282f01a8c

[2] Sato S, Kuwana M. Utility of dermatomyositis-specific autoantibodies for diagnosis and clinical subsetting. Int J Clin Rheumatol. 2015;10: 257–271.

[3] Sato S, Hirakata M, Kuwana M, Suwa A, Inada S, Mimori T, et al Autoantibodies to a 140-kd polypeptide, CADM-140, in Japanese patients with clinically amyopathic dermatomyositis. Arthritis Rheum. 2005;52: 1571–1576. 15880816 10.1002/art.21023

[4] Sato S, Hoshino K, Satoh T, Fujita T, Kawakami Y, Fujita T, et al RNA helicase encoded by melanoma differentiation-associated gene 5 is a major autoantigen in patients with clinically amyopathic dermatomyositis: association with rapidly progressive interstitial lung disease. Arthritis Rheum. 2009;60: 2193–2200. 10.1002/art.24621 19565506

[5] Fujikawa K, Kawakami A, Kaji K, Fujimoto M, Kawashiri S, Iwamoto N, et al Association of distinct clinical subsets with myositis-specific autoantibodies towards anti-155/140-kDa polypeptides, anti-140-kDa polypeptides, and anti-aminoacyl tRNA synthetases in Japanese patients with dermatomyositis: a single-centre, cross-sectional study. Scand J Rheumatol. 2009;38: 263–267. 10.1080/03009740802687455 19444719

[6] Hoshino K, Muro Y, Sugiura K, Tomita Y, Nakashima R, Mimori T. Anti-MDA5 and anti-TIF1-gamma antibodies have clinical significance for patients with dermatomyositis. Rheumatology (Oxford). 2010;49: 1726–1733. 20501546 10.1093/rheumatology/keq153

[7] Hamaguchi Y, Kuwana M, Hoshino K, Hasegawa M, Kaji K, Matsushita T, et al Clinical correlations with dermatomyositis-specific autoantibodies in adult Japanese patients with dermatomyositis: a multicenter cross-sectional study. Arch Dermatol. 2011;147: 391–398. 10.1001/archdermatol.2011.52 21482889

[8] Cao H, Pan M, Kang Y, Xia Q, Li X, Zhao X, et al Clinical manifestations of dermatomyositis and clinically amyopathic dermatomyositis patients with positive expression of anti-melanoma differentiation-associated gene 5 antibody. Arthritis Care Res (Hoboken). 2012;64: 1602–1610. 22623119 10.1002/acr.21728

[9] Chaisson NF, Paik J, Orbai AM, Casciola-Rosen L, Fiorentino D, Danoff S, et al A novel dermato-pulmonary syndrome associated with MDA-5 antibodies: report of 2 cases and review of the literature. Medicine (Baltimore). 2012;91: 220–228. 22732950 10.1097/MD.0b013e3182606f0b PMC3726263

[10] Chen Z, Cao M, Plana MN, Liang J, Cai H, Kuwana M, et al Utility of anti-melanoma differentiation-associated gene 5 antibody measurement in identifying patients with dermatomyositis and a high risk for developing rapidly progressive interstitial lung disease: a review of the literature and a meta-analysis. Arthritis Care Res (Hoboken). 2013;65: 1316–1324. 23908005 10.1002/acr.21985

[11] Labrador-Horrillo M, Martinez MA, Selva-O'Callaghan A, Trallero-Araguas E, Balada E, Vilardell-Tarres M, et al Anti-MDA5 antibodies in a large Mediterranean population of adults with dermatomyositis. J Immunol Res. 2014;2014: 290797 10.1155/2014/290797 24741583 PMC3987881

[12] Bohan A, Peter JB. Polymyositis and dermatomyositis (first of two parts). N Engl J Med. 1975;292: 344–347. 1090839 10.1056/NEJM197502132920706

[13] Sontheimer RD. Would a new name hasten the acceptance of amyopathic dermatomyositis (dermatomyositis sine myositis) as a distinctive subset within the idiopathic inflammatory dermatomyopathies spectrum of clinical illness? J Am Acad Dermatol. 2002;46: 626–636. 11907524 10.1067/mjd.2002.120621

[14] Aletaha D, Neogi T, Silman AJ, Funovits J, Felson DT, Bingham CO 3rd, et al 2010 Rheumatoid arthritis classification criteria: an American College of Rheumatology/European League Against Rheumatism collaborative initiative. Arthritis Rheum. 2010;62: 2569–2581. 10.1002/art.27584 20872595

[15] Tan EM, Cohen AS, Fries JF, Masi AT, McShane DJ, Rothfield NF, et al The 1982 revised criteria for the classification of systemic lupus erythematosus. Arthritis Rheum. 1982;25: 1271–1277. 7138600 10.1002/art.1780251101

[16] Subcommittee for Scleroderma Criteria of the American Rheumatism Association Diagnostic and Therapeutic Criteria Committee. Preliminary criteria for the classification of systemic sclerosis (scleroderma). Arthritis Rheum. 1980;23: 581–590. 7378088 10.1002/art.1780230510

[17] Vitali C, Bombardieri S, Jonsson R, Moutsopoulos HM, Alexander EL, Carsons SE, et al Classification criteria for Sjögren’s syndrome: a revised version of the European criteria proposed by the American-European Consensus Group. Ann Rheum Dis. 2002;61: 554–558. 12006334 10.1136/ard.61.6.554 PMC1754137

[18] Porter JF, Kingsland LC 3rd, Lindberg DA, Shah I, Benge JM, Hazelwood SE, et al The AI/RHEUM knowledge-based computer consultant system in rheumatology. Performance in the diagnosis of 59 connective tissue disease patients from Japan. Arthritis Rheum. 1988;31: 219–226. 3279963 10.1002/art.1780310210

[19] Watanabe K, Handa T, Tanizawa K, Hosono Y, Taguchi Y, Noma S, et al Detection of antisynthetase syndrome in patients with idiopathic interstitial pneumonias. Respir Med. 2011:105: 1238–1247. 10.1016/j.rmed.2011.03.022 21514811

[20] Kato H, Takeuchi O, Mikamo-Satoh E, Hirai R, Kawai T, Matsushita K, et al Length-dependent recognition of double-stranded ribonucleic acids by retinoic acid-inducible gene-I and melanoma differentiation-associated gene 5. J Exp Med. 2008;205: 1601–1610. 10.1084/jem.20080091 18591409 PMC2442638

[21] Nakashima R, Imura Y, Hosono Y, Seto M, Murakami A, Watanabe K, et al The multicenter study of a new assay for simultaneous detection of multiple anti-aminoacyl-tRNA synthetases in myositis and interstitial pneumonia. PLoS One. 2014;9: e85062 10.1371/journal.pone.0085062 24454792 PMC3891809

[22] Nakashima R, Imura Y, Kobayashi S, Yukawa N, Yoshifuji H, Nojima T, et al The RIG-I-like receptor IFIH1/MDA5 is a dermatomyositis-specific autoantigen identified by the anti-CADM-140 antibody. Rheumatology (Oxford). 2010;49: 433–440. 20015976 10.1093/rheumatology/kep375

[23] Tanizawa K, Handa T, Nakashima R, Kubo T, Hosono Y, Watanabe K, et al HRCT features of interstitial lung disease in dermatomyositis with anti-CADM-140 antibody. Respir Med. 2011;105: 1380–1387. 10.1016/j.rmed.2011.05.006 21632230

[24] Hall JC, Casciola-Rosen L, Samedy LA, Werner J, Owoyemi K, Danoff SK, et al Anti-MDA5-associated dermatomyositis: Expanding the clinical spectrum. Arthritis Care Res (Hoboken). 2013;65: 1307–1315. 23436757 10.1002/acr.21992 PMC3689861

[25] Fiorentino D, Chung L, Zwerner J, Rosen A, Casciola-Rosen L. The mucocutaneous and systemic phenotype of dermatomyositis patients with antibodies to MDA5 (CADM-140): a retrospective study. J Am Acad Dermatol. 2011;65: 25–34. 10.1016/j.jaad.2010.09.016 21531040 PMC3167687

[26] Koga T, Fujikawa K, Horai Y, Okada A, Kawashiri SY, Iwamoto N, et al The diagnostic utility of anti-melanoma differentiation-associated gene 5 antibody testing for predicting the prognosis of Japanese patients with DM. Rheumatology (Oxford). 2012;51: 1278–1284. 22378718 10.1093/rheumatology/ker518

[27] Ceribelli A, Fredi M, Taraborelli M, Cavazzana I, Tincani A, Selmi C, et al Prevalence and clinical significance of anti-MDA5 antibodies in European patients with polymyositis/dermatomyositis. Clin Exp Rheumatol. 2014;32: 891–897. 25151986

[28] Sato S, Kuwana M, Fujita T, Suzuki Y. Amyopathic dermatomyositis developing Rapidly progressive interstitial lung disease with elevation of anti-CADM-140/MDA5 autoantibodies. Mod Rheumatol. 2012;22: 625–629. 10.1007/s10165-011-0558-9 22124544

[29] Sato S, Kuwana M, Fujita T, Suzuki Y. Anti-CADM-140/MDA5 autoantibody titer correlates with disease activity and predicts disease outcome in patients with dermatomyositis and rapidly progressive interstitial lung disease. Mod Rheumatol. 2013;23: 496–502. 10.1007/s10165-012-0663-4 22644102

